# Carotid Artery Dissection and Hemorrhagic Stroke in the Setting of Multisystem Inflammatory Syndrome in Children

**DOI:** 10.7759/cureus.13640

**Published:** 2021-03-01

**Authors:** Irim Salik, Michael Jacoby

**Affiliations:** 1 Anesthesiology, Westchester Medical Center, Valhalla, USA

**Keywords:** non-traumatic carotid artery dissection, hemorrhagic stroke, pediatric anesthesiology

## Abstract

An alarming and distressing delayed inflammatory response to Coronavirus Disease 2019 (COVID-19) has emerged in children and adolescents, a population previously thought to have been largely spared by this global pandemic. In April 2020, clinicians in the United Kingdom identified eight cases of previously healthy children testing positive for current or recent infection with Severe Acute Respiratory Syndrome Coronavirus 2 (SARS-CoV-2) presenting with Kawasaki disease-like features. From April 16 to May 4, 2020, New York City reported that 15 patients aged 2-15 years were hospitalized with a similar multisystem inflammatory syndrome, many requiring intensive care unit (ICU) admission. This syndrome is referred to either as pediatric inflammatory multisystem syndrome, or multisystem inflammatory syndrome in children (MIS-C). We present the case of an eight-year-old with MIS-C who developed carotid artery dissection following venoarterial extracorporeal membrane oxygenation (VA-ECMO) cannulation, followed by a subsequent hemorrhagic stroke in the basal ganglia.

## Introduction

The pediatric population is affected much less commonly than adults with the Coronavirus Disease 2019 (COVID-19), with only 2% of cases described in patients younger than 20 years of age, and 90% of these cases classified as asymptomatic, mild, or moderate [1]. Classically, it is speculated that children have had milder presentations of COVID-19 as compared to adults due to immature development of the angiotensin-converting enzyme-2 receptor, as well as potential cross-immunity from other common viral infections [2]. Fortunately, the absolute number of children with the life-threatening multisystem inflammatory syndrome in children (MIS-C) is rare. There are few reports of neurologic sequelae in children following COVID-19 infection. Severe Acute Respiratory Syndrome Coronavirus 2 (SARS-CoV-2) may enter the central nervous system hematogenously, through the lymphatic system, the cerebral spinal fluid, or through the olfactory and trigeminal nerves [2]. With an underlying diagnosis of MIS-C, this patient developed severe neurologic complications following carotid artery dissection secondary to extracorporeal membrane oxygenation (ECMO) cannulation. The patient’s family provided authorization to publish this manuscript.

## Case presentation

**Figure 1:**
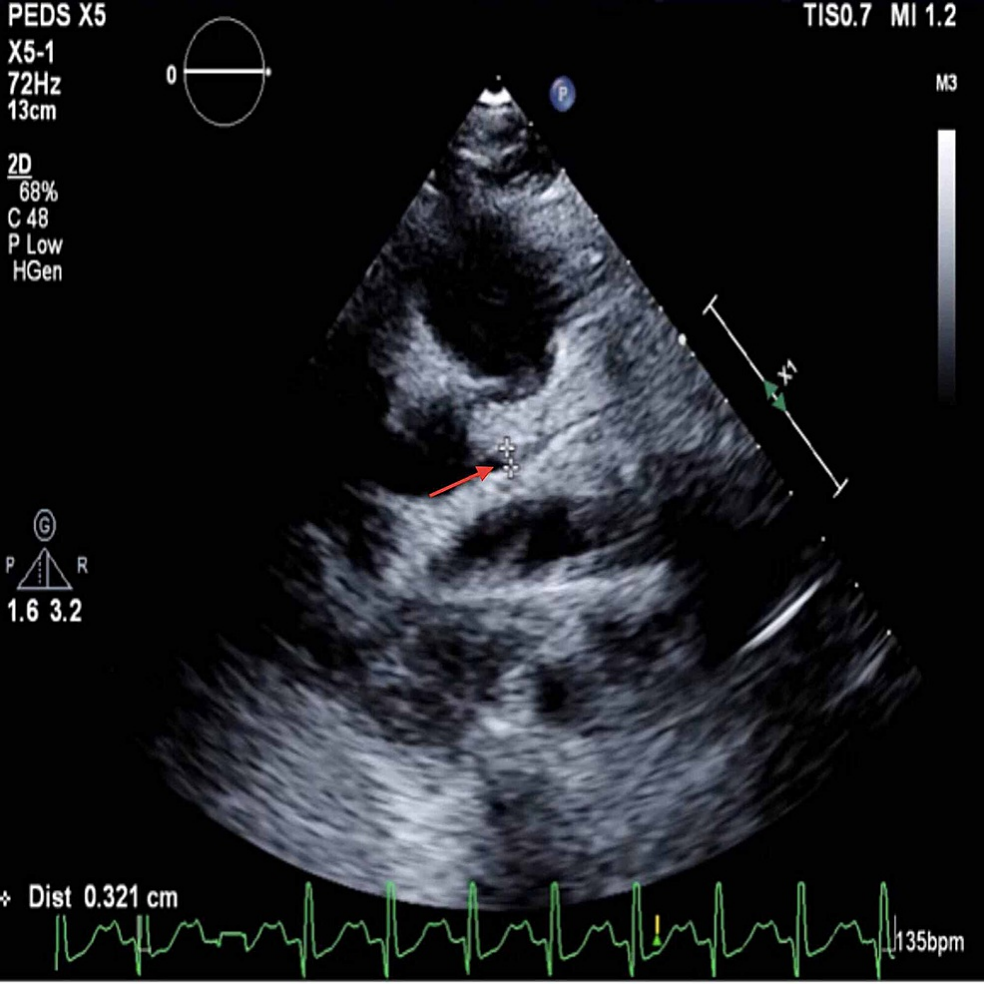
Dilated left main coronary artery

An eight-year-old male with no chronic medical conditions presented with a four-day history of fever, vomiting, erythematous rash, pedal edema and a two-day history of dyspnea. Pertinent lab findings on admission included lactate level of 4.1 mmol/L, brain natriuretic peptide (BNP) of 2095 pg/L, elevated D-Dimer at 8.75 mg/L, platelet count of 93,000/μL, and a troponin level of 0.35ng/mL which peaked to 0.8 ng/mL on hospital day (HD) 1. Nasopharyngeal SARS-CoV-2 polymerase chain reaction (PCR) testing was negative but the COVID-19 immunoglobulin (IgG) antibody test was positive. Both parents were sick with COVID-19 about four weeks prior and recovered uneventfully at home. Chest radiography demonstrated bilateral pulmonary infiltrates and echocardiogram revealed moderately dilated left main coronary, left anterior descending and left circumflex arteries, with an ejection fraction (EF) of 25% (Figure 1).

The patient was admitted to the pediatric intensive care unit (PICU) and started on intravenous immunoglobulin (IVIG) 2 g/kg, methylprednisolone 2 mg/kg/day, and anakinra for presumed COVID-19 cytokine storm on HD 2. The patient further decompensated in the PICU requiring intubation for depressed mental status and increasing lethargy. An epinephrine infusion was titrated for blood pressure maintenance in the setting of left ventricular hypokinesis on HD 3. Despite increasing vasoactive infusions, the patient became severely hypotensive and bradycardic and was treated with one round of pediatric advanced life support. Shortly thereafter, the patient was transitioned to VA-ECMO via right carotid artery and left femoral vein cannulation.

Despite full ECMO support, the patient exhibited elevated left atrial pressures, rising lactate and BNP levels, and began to develop pulmonary edema on HD 3. He developed acute renal failure which required continuous venovenous hemodialysis via the ECMO circuit. His echocardiogram revealed an EF of 10-15%. Interventional cardiology was consulted to perform an atrial septostomy in the cardiac catheterization lab to offload the left atrium, which proceeded uneventfully. Following intervention in the catheterization lab, cardiac function improved on HD 5. The patient was weaned off ECMO on HD 7 and remained on milrinone and dobutamine infusions until HD 8.

**Figure 2:**
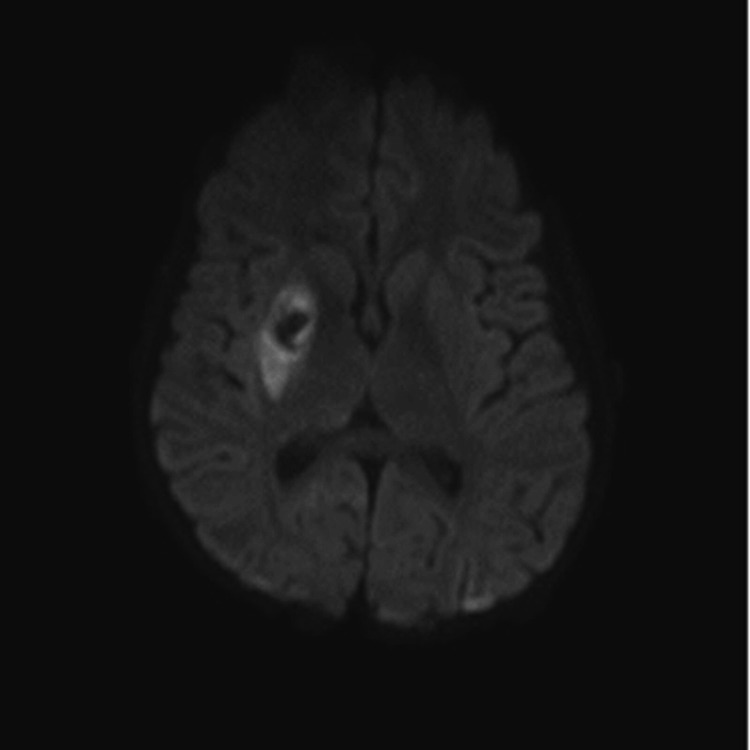
MRI image of ischemic infarct of right basal ganglia with hemorrhagic transformation

As sedation was weaned in an attempt to extubate the patient, depressed mental status persisted. Magnetic resonance imaging revealed a right carotid artery dissection and thrombus, presumably as a result of ECMO cannulation. In addition, an ischemic infarct was noted in the right basal ganglia with hemorrhagic transformation (Figure 2). A neurology consult attributed this to vascular injury and an underlying hypercoagulable state secondary to MIS-C. The patient was started on therapeutic enoxaparin 1 mg/kg. Furosemide was initiated to promote diuresis, and coincided with further improvement of cardiac and renal function. Repeat echocardiogram revealed an EF of 55% and the patient was extubated on HD 9. Mental status gradually improved throughout the hospital stay, although the patient exhibited residual left-sided hemiparesis. He was discharged to a pediatric long-term care facility on HD 20.

## Discussion

MIS-C is a novel syndrome in the pediatric population that is temporally related to SARS-CoV-2 exposure. It shares a number of features with other pediatric inflammatory conditions including Kawasaki disease (KD), staphylococcal and streptococcal toxic shock syndromes, sepsis, and macrophage activation syndrome, including skin rash, lymphadenopathy, diarrhea and inflammatory marker elevation. MIS-C is defined by presentation of fever, laboratory evidence of inflammation (elevated C-reactive protein [CRP], erythrocyte sedimentation rate, fibrinogen, procalcitonin, d-dimer, ferritin, lactic acid dehydrogenase or interleukin 6 (IL-6), elevated neutrophils, reduced lymphocytes and low albumin) with multisystem organ involvement and recent positive SARS-CoV-2 infection by reverse transcriptase PCR, serology, or antigen test [3]. Our patient exhibited cardiac and gastrointestinal symptoms in addition to fever and lactic acidosis in the setting of SARS-CoV-2 exposure.

A case series by Chiotos et al. distinguishes MIS-C from KD based upon troponin leak and grossly elevated BNP, frequent and severe enteropathy, and thrombocytopenia [4]. Cardiac involvement and acute heart failure are likely due to myocardial stunning or edema as opposed to an inflammatory response. Macrophage activation along with stretched cardiomyocytes leads to grossly elevated IL-6 levels, subsequently leading to vasoplegia [5]. Fever and gastrointestinal symptoms often precede the presentation of classic features of KD. Whittaker et al. have described three patterns of disease amongst hospitalized children with MIS-C: first with persistent fever and elevated levels of inflammatory markers but without features of KD, organ failure or shock; the second group exhibited diagnostic criteria for KD; and a third group presented with shock and clinical, laboratory, and echocardiographic evidence of myocardial injury [6].

A study by Toubiana et al. describes 21 children and adolescents in France presenting with Kawasaki-like disease, most of whom were of African or Caribbean ancestry [7]. All patients presented with leukocytosis and elevated inflammatory markers, primarily CRP and serum IL-6; two patients exhibited electrocardiographic changes and arrhythmias. All patients were treated with aspirin and IVIG, while half the sample received corticosteroids. Even though 81% of patients required ICU care, with 71% relying on inotropic agents and 52% receiving mechanical ventilation, all patients survived. Children presented about four weeks after widespread lockdowns, with a median of 36 days after contact with a person with known or presumed COVID-19. The delay between the peak of SARS-CoV-2 infection and acute presentation of MIS-C gives credence to the theory that this is a post-infectious, immunologically mediated phenomenon of COVID-19.

Venoarterial ECMO (VA-ECMO) is utilized in patients with severe cardiac failure refractory to medical management. In pediatrics, central cannulation via the mediastinum is accompanied by peripheral cannulation via the right internal jugular vein and the right common carotid artery. Although carotid artery dissection in the context of VA-ECMO is uncommon, it has been previously described [8]. In the acute setting of dissection, antithrombotic and antiplatelet therapy should be continued. Surgical intervention is reserved for patients with significant complications, including severe stenosis or recurrent emboli, as it can increase the risk of early occlusion, stroke, and cranial nerve injuries [9]. Endovascular modalities are also a safe option in patients, but may not provide any advantage over medical therapy alone.

Carotid artery cannulation is independently associated with the risk for neurologic injury in the pediatric population. There is a potential risk for derangement of cerebral autoregulation, impairment of cerebral venous drainage, increased risk of cerebral embolic phenomena, intracerebral hemorrhage (ICH), seizures and anoxic injuries [9]. VA-ECMO leads to a higher risk for neurologic morbidity than veno-venous ECMO due to the severity of the patient’s underlying cardiopulmonary dysfunction, loss of pulsatile flow, and circuit-associated emboli [10]. In patients who suffer carotid artery dissection, the decision to repair or ligate the artery is based upon concern for compromised cerebral perfusion through the Circle of Willis. Repair of the carotid artery is not to be taken lightly, as it may be associated with stroke, anastomotic dehiscence, and aneurysm formation [11].

The most common neurologic complications following ECMO cannulation include embolic ischemic strokes, watershed infarctions, ICH, seizures, brain death and diffuse cerebral edema, followed by prolonged coma and hypoxic-ischemic encephalopathy [12]. During ECMO support, a significant reduction in cerebral blood flow (CBF) is common, as evidenced by reduced mean flow velocities on transcranial Doppler sonography. The flow pattern is characterized by decreased systolic upstroke, the disappearance of the dicrotic notch, and continuous diastolic flow. Reduced CBF can be attributed to decreased cerebral metabolic rate due to sedative medication use, cerebral venous congestion due to jugular vein dilation, and left ventricular dysfunction in patients supported by VA-ECMO [13]. Patients who develop ICH secondary to ECMO are more likely to experience reactive hyperemia with increases in CBF two to six days prior to clinical recognition of neurologic injury, due to CBF uncoupling [12]. In a patient with COVID-19, the use of anticoagulants, recent ischemic injury, and an underlying coagulopathic state may greatly increase the risk of ICH.

## Conclusions

In the setting of MIS-C, a patient’s increased inflammatory response and elevated acute-phase reactants may result in hypercoagulability and thrombotic events. Embolic areas that become ischemic may be prone to hemorrhagic transformation. In the face of the patient’s underlying thrombocytopenia, impaired platelet function, fibrinolysis, clotting factor consumption and therapeutic anticoagulation secondary to ECMO cannulation, there was an increased likelihood of hemorrhagic complications. Practitioners must be vigilant of such devastating complications in patients with MIS-C who require ECMO cannulation following cardiac decompensation.

## References

[1] (2020). Centers for Disease Control and Prevention. Multisystem Inflammatory Syndrome in Children (MIS-C) associated with Coronavirus Disease 2019 (COVID-19). https://emergency.cdc.gov/han/2020/han00432.asp.

[2] Dong Y, Mo X, Hu Y, Qi X, Jiang F, Jiang Z, Tong S (2020). Epidemiology of COVID-19 among children in China. Pediatrics.

[3] Li Z, Liu T, Yang N (2020). Neurological manifestations of patients with COVID- 19: potential routes of SARS-CoV-2 neuroinvasion from the periphery to the brain. Front Med.

[4] Chiotos K, Bassiri H, Behrens EM (2020). Multisystem Inflammatory Syndrome in Children during the COVID-19 pandemic: a case series. J Pediatric Infect Dis Soc.

[5] Belhadjer Z, Méot M, Bajolle F (2020). Acute heart failure in multisystem inflammatory syndrome in children (MIS-C) in the context of global SARS-CoV-2 pandemic. Circulation.

[6] Whittaker E, Bamford A, Kenny J (2020). Clinical characteristics of 58 children with a pediatric Inflammatory Multisystem Syndrome temporally associated with SARS-CoV-2. JAMA.

[7] Toubiana J, Poirault C, Corsia A (2020). Kawasaki-like disease multisystem inflammatory syndrome in children and adolescents during the covid-19 pandemic in Paris, France: prospective observational study. BMJ.

[8] Ryerson LM, Sanchez-Glanville C, Huberdeau C, Aklabi MA (2017). Carotid artery dissection following neck cannulation for extracorporeal life support. World J Pediatr Congenit Heart Surg.

[9] Teele SA, Salvin JW, Barrett CS (2014). The association of carotid artery cannulation and neurologic injury in pediatric patients supported with venoarterial extracorporeal membrane oxygenation. Pediatr Crit Care Med.

[10] Polito A, Barrett CS, Wypij D (2013). Neurologic complications in neonates supported with extracorporeal membrane oxygenation. an analysis of ELSO registry data. Intensive Care Med.

[11] Duggan EM, Maitre N, Zhai A (2015). Neonatal carotid repair atECMO decannulation: patency rates and early neurologic outcomes. J Pediatr Surg.

[12] Bowling SM, Gomes J, Firstenberg MS (2015). Neurologic issues in patients receiving extracorporeal membrane oxygenation support. Extracorporeal Membrane Oxygenation - Advances in Therapy.

[13] Raju TN, Kim SY, Meller JL (1989). Circle of Willis blood velocity and flow direction after common carotid artery ligation for neonatal extra-corporeal membrane oxygenation. Pediatrics.

